# The Effects of CO_2_ Laser with or without Nanohydroxyapatite Paste in the Occlusion of Dentinal Tubules

**DOI:** 10.1155/2014/798732

**Published:** 2014-10-20

**Authors:** Mohammed Abbood Al-maliky, Ali Shukur Mahmood, Tamara Sardar Al-karadaghi, Christoph Kurzmann, Markus Laky, Alexander Franz, Andreas Moritz

**Affiliations:** ^1^Division of Conservative Dentistry and Periodontology, Bernhard Gottlieb University Clinic of Dentistry, Medical University of Vienna, Sensengasse 2a, 1090 Vienna, Austria; ^2^Department of Biomedical Applications, Institute of Laser for Postgraduate Studies, University of Baghdad, Aljadriya Campus, Baghdad, Iraq; ^3^Division of Dental Student Training and Patient Care, Bernhard Gottlieb University Clinic of Dentistry, Medical University of Vienna, Sensengasse 2a, 1090 Vienna, Austria; ^4^Central Research Unit and Division of Conservative Dentistry, Bernhard Gottlieb University Clinic of Dentistry, Medical University of Vienna, Sensengasse 2a, 1090 Vienna, Austria

## Abstract

The aim of this study was to evaluate a new treatment modality for the occlusion of dentinal tubules (DTs) via the combination of 10.6 *µ*m carbon dioxide (CO_2_) laser and nanoparticle hydroxyapatite paste (n-HAp). Forty-six sound human molars were used in the current experiment. Ten of the molars were used to assess the temperature elevation during lasing. Thirty were evaluated for dentinal permeability test, subdivided into 3 groups: the control group (C), laser only (L−), and laser plus n-HAp (L+). Six samples, two per group, were used for surface and cross section morphology, evaluated through scanning electron microscope (SEM). The temperature measurement results showed that the maximum temperature increase was 3.2°C. Morphologically groups (L−) and (L+) presented narrower DTs, and almost a complete occlusion of the dentinal tubules for group (L+) was found. The Kruskal-Wallis nonparametric test for permeability test data showed statistical differences between the groups (*P* < 0.05). For intergroup comparison all groups were statistically different from each other, with group (L+) showing significant less dye penetration than the control group. We concluded that CO_2_ laser in moderate power density combined with n-HAp seems to be a good treatment modality for reducing the permeability of dentin.

## 1. Introduction

Dentin hypersensitivity (DH) arises from exposed dentin in response to tactile, thermal, osmotic, evaporative, and chemical stimuli, which cannot be attributed to any other form of dental defect or pathology. DH is characterized by short, sharp pain and is often faced as a bothering symptom in dental clinics [1]. Basically, exposure of the dentin results from one of two processes, either removal of the enamel covering the crown of the tooth or denudation of the root surface by loss of cement and overlying periodontal tissues [2, 3].

Hypersensitive teeth have a larger number and wider diameter of exposed DTs than normal teeth. That is the reason why treatment modalities often focus on decreasing the radius of the open dentinal tubules (DTs) [4]. Scanning electron microscopic (SEM) examinations of human DTs showed a number of approximately 20,000/mm^2^ at the surface of peripheral dentin [5]. Isik et al. [6] stated that, on untreated dentin, the diameter of DTs ranges from 1.76 to 2.12 *μ*m.

In 2006, Bartold [7] postulated that 14.3% of all patients have some degree of sensitivity. Incidence peaks around the third decade of life with no gender preference [8, 9]. Maxillary premolars are the most commonly affected teeth and cold drinks are most often the triggering factor [10, 11]. DH prevalence seems to range from 60% to 98% in patient with periodontitis [12]. So dentin hypersensitivity is a widespread and common problem.

In spite of extensive research, DH mechanism and management are still only partially understood. One mechanism in the treatment of DH is hydrodynamic one; here the treatment focus is on desensitizing agents and dentifrices mainly containing fluorides that have the ability to seal or occlude the DTs through calcium fluoride crystal precipitation. Another one is neural, by decreasing the activity of the dentinal sensory nerve. Potassium nitrate is mainly used for this mechanism. Until now, no treatment certainly eliminates DH [10, 13].

In 1935, Grossman [14] listed the basic requirements that an ideal dentifrice or desensitizer should have, which are still valid now: nontoxic material, not irritating the pulp, being easy to apply and spread, being consistently effective, being permanently active, and having rapid performance, and it should not cause tooth discoloration. Considering possible materials to use for this purpose, nanohydroxyapatite (n-HAp) could be a good option because of its similar composition to tooth and bone. It is a widely accepted material in dentistry and in medicine, because it has a very high level of biocompatibility and bioactivity [15, 16]. n-HAp particle diameter is in the nanometer scale, which is much smaller than that of the DTs. In general, surface area and chemical reactivity of the material increase with decreasing particle size. Assuming equal masses of nano- and micrometer particle diameter of the same material, the surface area and chemical reactivity are approximately 1000-fold greater [17].

Since the invention of the laser by Maiman [18] in 1960, researchers have investigated laser applications in dentistry, and since that time, lasers have added additional revolutionary treatment options for both hard and soft tissue applications in dentistry.

The mechanisms by which lasers act on tissue depend on factors of the tissue itself and the laser parameters. Lasers have also been used in the treatment of DH. High output power laser systems such as neodymium: yttrium-aluminum-garnet (Nd:YAG), erbium: yttrium-aluminum-garnet (Er:YAG), and carbon dioxide (CO_2_) can decrease or even eliminate dentinal pain due to their ability to occlude DTs [19, 20]. Diode laser achieved good results in DH treatment through causing stenosis of the DTs and reduced dye penetration across the dentin [21]. Pashley et al. [22] reported that CO_2_ laser irradiation is able to occlude DTs and decrease dentinal permeability by reducing hydraulic conductance.

The selective absorption of 10.6 *μ*m CO_2_ laser by hydroxyapatite paste makes this wavelength the appropriate candidate for treatment of DH. To our knowledge, there are no studies addressing the combination of a CO_2_ laser and a nanoparticle hydroxyapatite paste with this extremely small particle size. This small particle size possesses the ability to enter inside the DTs to increase [23] its surface area and to promote its absorption onto tooth surfaces.

This study investigates in vitro the effects of CO_2_ laser with or without n-HAp on dentin permeability, temperature elevation, and morphology.

## 2. Materials and Methods

### 2.1. Sample Preparation

**Figure 1:**
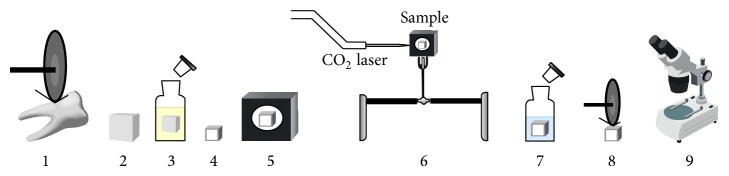
Permeability test setup and method. 1: tooth sectioning. 2: samples collection and cementum removal. 3: immersion in 1% citric acid for 5 min. 4: marking of the lasing area and nail varnish coating. 5: mounting in acrylic resin mold. 6: lasing, 7: immersion in 2% methylene blue for 1 hr. 8: longitudinal sectioning. 9: stereomicroscopic examination.

In this study 46 sound extracted human molars were selected (due to periodontal indications) after approval of the Ethics Review Committee of the Medical University of Vienna on research (EK Nr: 980/2009). Thirty molars were used for the permeability test, six samples for the SEM examination, and the last ten teeth for temperature measurement. After teeth apices were mounted in acrylic resin, two horizontal sections were made using a diamond saw blade (915 DC, Meisinger, Germany), which is mounted on a low speed hand piece (W&H A 25 RM, Dabi Atlante, Austria) under running distilled water. One section was done at the cement-enamel junction and the second 3 mm apical to the first one. The cementum was removed by a periodontal curette (4L-4R, GC-AMERICAN, USA) before the second sectioning was done. Samples of 3 × 4 mm area were obtained from each molar. The teeth were immersed in 1% citric acid solution for 5 minutes [24] for smear layer removal, then washed with distilled water in an ultrasonic bath for 15 minutes, and dried with gauze (Figure 1).

The surface and cross section morphology of all samples were evaluated using SEM (Tabletop Microscope TM-1000, Hitachi high technologies corp., Tokyo, Japan). Six samples were used; three of them were utilized for assessment of surface morphology and the other three samples were used to examine the cross section morphology after different treatment modalities. For the cross section examination, the three samples were fractured with a dental chisel after making a groove at the pulpal side of the sample opposite to the lasing side, with the aid of a diamond bur mounted on a high speed air motor with water spray.

**Figure 2:**
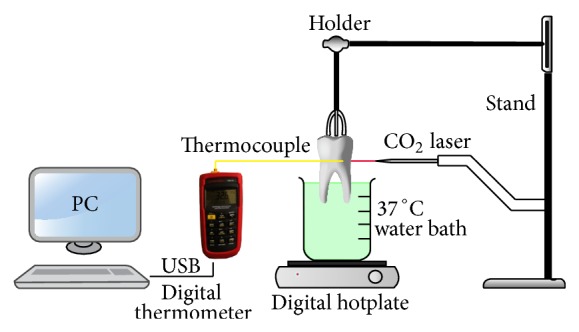
Experimental setup for temperature measurement.

A whole tooth was used for the temperature measurement experiment. The lasing was done after cementum removal and marking an area of 3 × 4 mm for lasing. A hole was made with a diamond fissure bur in the root surface opposite to the lasing area, until reaching the pulp cavity. The teeth were mounted in acrylic resin at the crown portion (Figure 2).

### 2.2. Samples Grouping and Treatment

Thirty-six exposed DTs samples were divided into three groups (*n* = 12) (Table 1), the control (C), laser (L−), and laser plus n-HAp (L+), two per group for the SEM and ten for each group for the permeability test. The (C) group samples received no treatment after surface conditioning with 1% citric acid. For the group (L−) the samples were irradiated with a CO_2_ laser only, and for the (L+) group the CO_2_ laser irradiation was done after applying the n-HAp paste (M K Impex Corp., Ontario, Canada). With an average particle size of 60 nm, the paste was prepared by mixing the n-HAp powder with distilled water, and then it was added to the dentin surface by a microbrush 10 times over a period of 10 minutes with hand pressure. After that, excess paste was brushed away. The samples were irradiated with a CO_2_ laser (OpusDuo EC, Lumenis Germany GmbH), 10.6 *μ*m wavelength, 0.65 W, in a continuous mode perpendicular to dentin surface, with 5 mm defocus distance, and a power density of (129.33 W/cm^2^). The lasing was done according to Moritz et al. [20], 6 times for 5 s with 20 s interval for cooling in between. After treatment, the samples were rinsed with a distilled water spray for 15 seconds.

### 2.3. Dye Penetration Test

The specimens were coated with three layers of nail varnish except in the marked area and then were immersed in an aqueous solution of 2% methylene blue dye for 1 hour at room temperature. The samples were then washed under tap water, dried, and cut longitudinally and the cross section of each sample was examined with a stereomicroscope (Hamilton, Altay Scientific, Rome, Italy).

The permeability test was evaluated by using the measure pictures V 1.0 software (CAD-KAS Kassler Computer software GbR, Germany). A stereomicroscope under the magnification of ×50 was used to measure the length of dye penetration in the DTs from the outer surface of the root toward the pulp chamber. For the standardization of the samples measurements, the length of dye penetration inside the dentin was divided by the whole thickness of the sample. Then the results multiply by 100%.

### 2.4. Temperature Measurement

The measurements were done opposite to the lasing area under the following conditions. Part of the root was immersed in water, which was heated by an accurate digital hotplate (Cemaric^*^ , Thermo Scientific Inc., MA, USA) for teeth temperature stabilization at 37 ± 0.5°C while being immersed in water. A K type thermocouple was used, which was connected to a digital multilogger thermometer (Amprobe TMD-56, Everett, WA, USA), with basic accuracy of ±0.05%, through a universal serial bus controller connected to a computer software (Amprobe multiline V3.0). The temperature recording was done every second. A thermal compound of 5.6 W/MK thermal conductivity (Arctic MX-2, China) was injected inside the pulp chamber, to confirm a contact between the thermocouple and the dentin surface during temperature measurements. Then a horizontal tooth sectioning through the premarked lasing area was done to measure the thickness of all samples by a vernier caliper (TOPEX Sp. z o.o. S.K., Warsaw, Poland), from the peripheral dentin surface to the pulpal one.

## 3. Results

### 3.1. Dye Penetration Test

The data obtained from dye penetration from all the experimental groups were statistically analyzed using SPSS Statistics 20 (IBM Corp., NY, USA). Descriptive statistics were done to obtain the means and standard deviations (Table 2).

Group (C) showed an obvious dye penetration approaching the pulpal side of the dentin with a mean of 86.52%, while (L+) exhibited only a slight penetration of the dye beyond the dentin surface with a mean of 16.22%. In group (L−), the mean was 49.39% which seemed to have dye penetration in the DTs.

**Figure 3:**
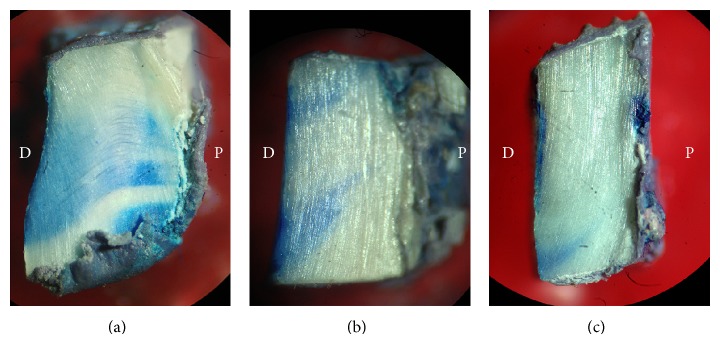
Dye penetration test. D: dentin surface side; P: pulpal side. (a) Control group (C), (b) laser only (L−), and (c) the laser plus nanohydroxyapatite paste (L+). The (C) group shows a full length dye penetration while for the (L+) group the dye is confined mainly to the surface with very slight dye penetration.

To check if the obtained data distribution is normal, the Shapiro-Wilk test was implemented, and the test statistics showed that data were not normally distributed (*P* < 0.05). To examine if the groups were statistically different, the Kruskal-Wallis nonparametric test was used and the obtained descriptive level was 0.002, which revealed that the groups were significantly different. For intergroups comparisons the post hoc Dunnett multiple comparison test was performed. The group (L+) possessed highly significant less dye penetration compared to the control group (C) (*P* < 0.001) (Figure 3); (L+) and (L−) groups were also statistically different (*P* < 0.05). Finally the laser group was significantly different compared to the control group (C) (*P* < 0.05).

### 3.2. SEM Evaluation

**Figure 4:**
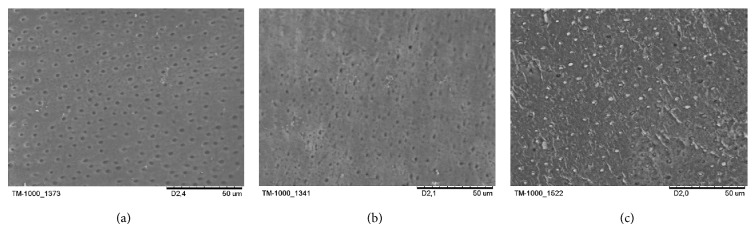
Dentine surface scanning electron microscope (SEM) micrographs. (a) Control group (C), (b) laser only (L−), and (c) the laser plus nanohydroxyapatite paste (L+). The original magnification was ×1200.

**Figure 5:**
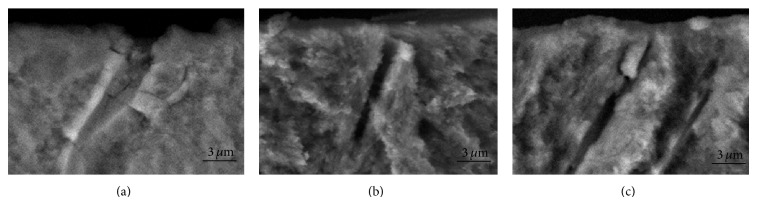
Cross-sectional scanning electron microscope (SEM) micrographs. (a) Control group (C), (b) laser only (L−), and (c) the laser plus nanohydroxyapatite paste (L+). The original magnification was ×5000.

The SEM showed the morphological characteristics of all different groups of the experiment. In group (C), the DTs were widely open and the whole area was free from smear layer (Figures 4(a) and 5(a)). The specimens of group (L−) (Figure 4(b)) showed a smaller diameter of DTs which is also noted in the cross section micrograph (Figure 5(b)). A large number of DTs were occluded due to the melting effect of the CO_2_ laser. In the combined group (L+) (Figure 4(c)), most tubules were occluded by n-HAp, and there were signs of melting of the n-HAp. The cross section micrograph of the combined group (L+) (Figure 5(c)) showed melting of the n-HAp, penetration into the tubules, and formation of plug over it.

### 3.3. Temperature Measurements

**Figure 6:**
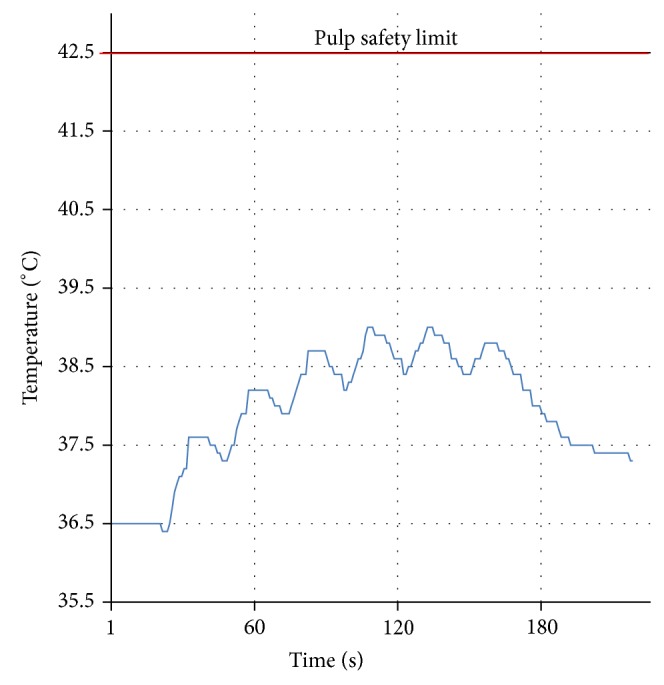
Temperature curve. A temperature increase of 2.5°C for the most representative sample. The six cycles of lasing are clearly visible.

Laser irradiation showed a mean temperature rise of 2.33°C ± 0.56°C, and the maximum temperature rise was 3.2°C. The corresponding average dentin thickness was 2.19 mm ± 0.24 mm. The curve of the temperature elevation course (Figure 6) showed a temperature increase of 2.5°C, revealing a favorable temperature drop in the 20-second interval between each 5-second lasing cycle.

## 4. Discussion

Various studies have been conducted to evaluate the effect of n-HAp on enamel and dentin remineralization [25, 26]. In this study we are trying to assess the effectiveness of this material in the treatment of DH. In this work, the ability of a CO_2_ laser to melt the nanoparticle hydroxyapatite paste and fuse the open DTs was examined regarding the morphology and permeability of dentin. Additionally, temperature measurements were performed.

The results of the three groups in this study showed that (L+) group has the greater decrease in dentin permeability. This outcome was supported by SEM as most of DTs were sealed by n-HAp plugs.

The (L+) group reduced the permeability more than (L−). This may indicate that the addition of n-HAp was effective in reducing dentinal permeability. In the (L−) group dye penetration inside the dentin was 49%. These results can be compared to Matsui et al. [21] who recorded a 41% dye extension inside the tooth dentin thickness. Bonin et al. [27] reported a reduction in the dentinal permeability with 1 W using CO_2_ laser alone. In the present study the n-HAp paste and the CO_2_ laser with 0.65 W proved to reduce the dentinal permeability to an acceptable level at a lower power setting.

In this study, 1% citric acid was used to remove the smear layer leaving a clean surface free of tubules plugs and without surface damage (Figure 4(a)). Citric acid is one of the agents used to remove the smear layer from the dentin surface [28, 29] which may simulate DH in vivo conditions due to its presence in juices, vegetables, and fruits [30]. Samples were stored without adding any antiseptics, as they may affect the dentin permeability due to mineral trapping inside the DTs [31].

**Figure 7:**
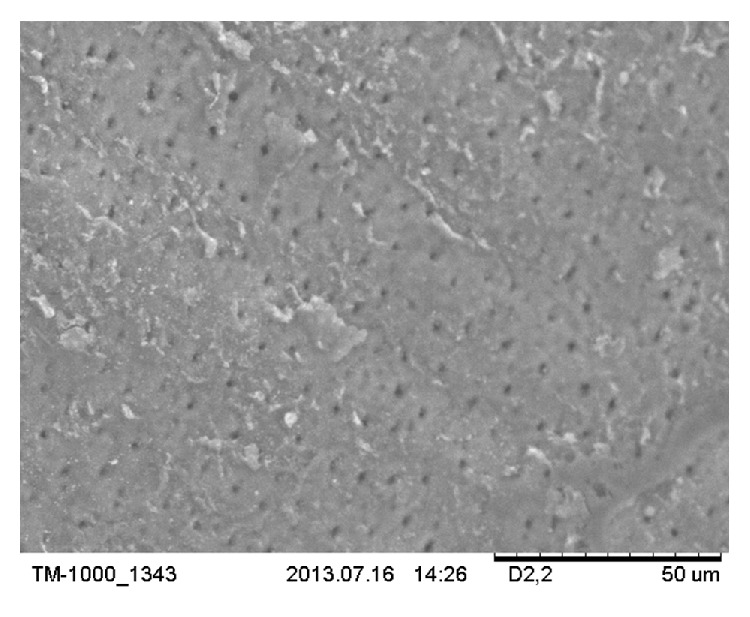
SEM micrograph of a pilot sample in which n-HAp only is applied on DTs. Showing open DTs like the SEM of the control group (C) with some remnant of the n-HAp paste remaining on the dentin surface. The original magnification was ×1200.

On SEM micrographs, for group (L+) most of the DTs were occluded. The n-HAp particles appeared to be melted, recrystallized, and trapped inside the tubules forming a plug inside their orifices (Figure 5(c)). These plugs resisted the 15 s air-water spray that was done after the lasing, which remained bonded to the DTs (Figure 4(c)). This may prove the ability of the CO_2_ laser in melting and bonding the n-HAp particles to the DTs. In pilot experiments of this study SEM shows that adding n-HAp paste only did not resist the 15 s air-water spray. Leave open DTs like the SEM of the control group (C) with some remnant of the n-HAp paste remaining on the dentin surface (Figure 7). The control group was chosen to be representative of open DTs. We may say that the combined treatment or the indirect method as first stated by Moritz et al. [20, 32] is a step forward to reduce the shortcoming of either treatment alone [23, 24, 33].

As shown in the (L−) micrograph (Figure 4(b)), dentin surface melting and narrowing of DTs diameter had been also noted. Similar results were presented by Cakar et al. [24] demonstrating a reduction of DTs diameter after exposure to CO_2_ laser. In the (L+) group micrograph, the melting of n-HAp may be due to the nanometer particles size of the n-HAp which reveals a large surface area promoting absorption on the dentin surface [23].

We measured the temperature to ensure that our parameter is within the pulp safety limit of a 5.5°C temperature increase as reported by Zach and Cohen [34]. The maximum recorded value was a temperature increase of 3.2°C inside the pulp chamber, with an average dentin thickness of 2.19 mm. This result is in agreement with Moritz et al.'s [20] who recorded a 2.5°C as maximum temperate increase. As the heat dissipated to the surrounding medium, the air or the water [35], we made a hole opposite the lasing area, so that the thermocouple inserted inside the pulp chamber was isolated by the tooth structure and may not detect the heat dissipated to that medium. We used a whole tooth instead of sectioned dentin samples for temperature measurement, such as what we had done in the permeability test. According to the basic law of thermodynamics, *dQ* = *mcdT*, where *dQ* is the heat content, *m* represents tooth mass, *c* is the heat capacity, and *dT* is the linear change in the temperature. The sample with a smaller mass may exhibit a higher temperature after the lasing procedure. A thermal compound was used inside the pulp chamber to prevent a gap formation between the thermocouple and the dentin.

According to the manufacturer specifications, the n-HAp used in this study is water insoluble. This may give favorable expectations, and while Romano et al. [35] used the calcium hydroxide paste in combination with CO_2_ laser, they stated that the stability of this paste is still a point for open discussion due to the solubility of this material.

Due to its high absorption in both hydroxyapatite and water, the CO_2_ laser with a wavelength of 10.6 *μ*m has high absorption coefficient in dentin (800 cm^−1^), presenting satisfactory superficial interaction. This kind of interaction is required for sealing DTs to reduce fluid passage across the dentin and consequently DH pain relief [35, 36].

Due to the presence of the dental pulp in vivo, this work needs to be confirmed clinically. The presence of pulp circulation in vivo may be of advantage in reducing the temperature elevation due to lasing as it acts as a heat sink that dissipates the generated heat [37]. Also, odontoblast cells in the dental pulp of primates and dogs could be indirectly activated by low power CO_2_ laser leading to reactional dentinogenesis [38, 39].

DH treatment by laser seems to be simple, quick, and effective [21]. He et al. [40] concluded through his systematic review that laser treatment with correct and controlled parameters will not lead to adverse effects. Our experiment with 0.65 W of moderate power density showed that there was no damage, carbonization, or cracks on the dentin surface, which is in accordance with previous studies employed for DH treatment [32, 35, 41].

This experiment showed that n-Hap, melted and plugged over most of the DTs, reduced dentinal permeability with an acceptable temperature increase using a CO_2_ laser of 0.65 W with a moderate power density.

## 5. Conclusions

Based on the results of the present study, the combination of nanohydroxyapatite paste and a CO_2_ laser of moderate power density occluded the dentinal tubules and reduced the permeability of exposed dentin. This preliminary experiment gives primary evidence of a new treatment modality for dentin hypersensitivity. Further comprehensive clinical studies are needed to assess the clinical potential of this combined treatment.

## Figures and Tables

**Table 1:** Sample grouping and treatment.

Groups	Laser	n-HA paste	Time (s)	Interval (s)	Power (W)	Spot size (mm)	Pd^a^ (W/cm²)
C^b^	—	—	—	—	—	—	—
L−^c^	CO_2_	−	6X (5 s)	5X (20 s)	0.65	0.4	129.33
L+^d^	CO_2_	+	6X (5 s)	5X (20 s)	0.65	0.4	129.33

^a^Power density.

^b^Control group.

^c^Laser only group.

^d^Laser plus nanoparticle hydroxyapatite paste group.

**Table 2:** Mean of percentage of dye penetration and standard deviation in each group.

Groups	Mean	Std. deviation
C^a^	86.52%	20.10
L−^b^	49.39%	43.29
L+^c^	16.22%	23.47

^a^Control group.

^b^Laser only group.

^c^Laser plus nanoparticle hydroxyapatite paste group.
